# Supplementary figures and images for: Pharmacological inhibition of CLK2 activates YAP by promoting alternative splicing of AMOTL2
Source: eLife. 2023 Dec 21;12:RP88508. doi: 10.7554/eLife.88508 (PMC10735217; doi:10.7554/eLife.88508)

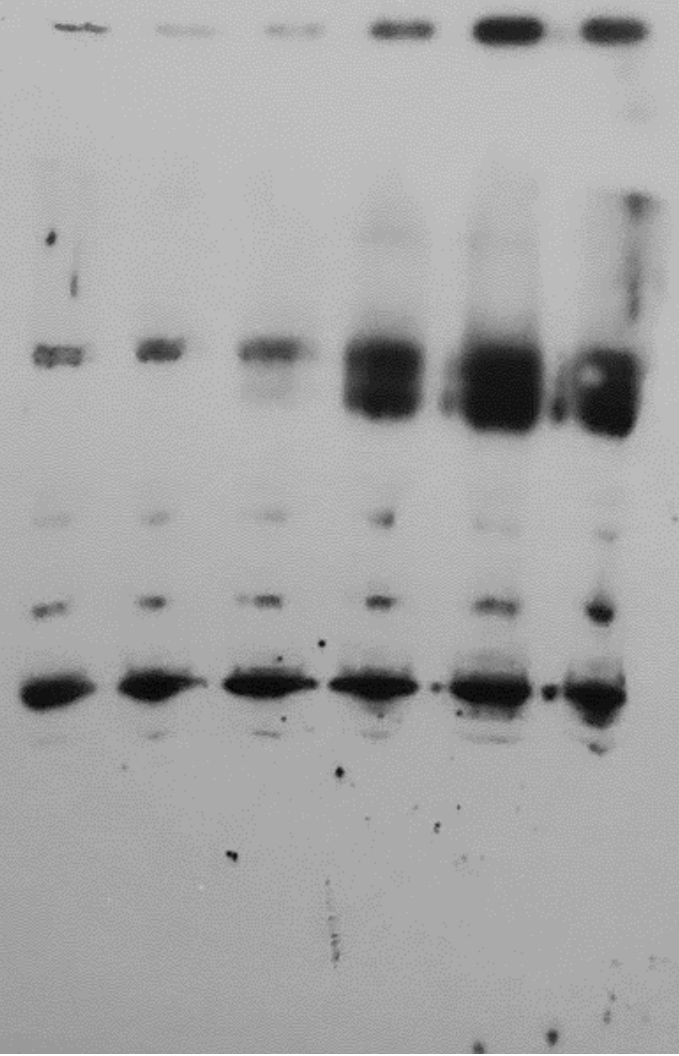

Supplement: Figure 3—source data 2. [file elife-88508-fig3-data2.zip › Figure 3D AMOTL2.pdf]

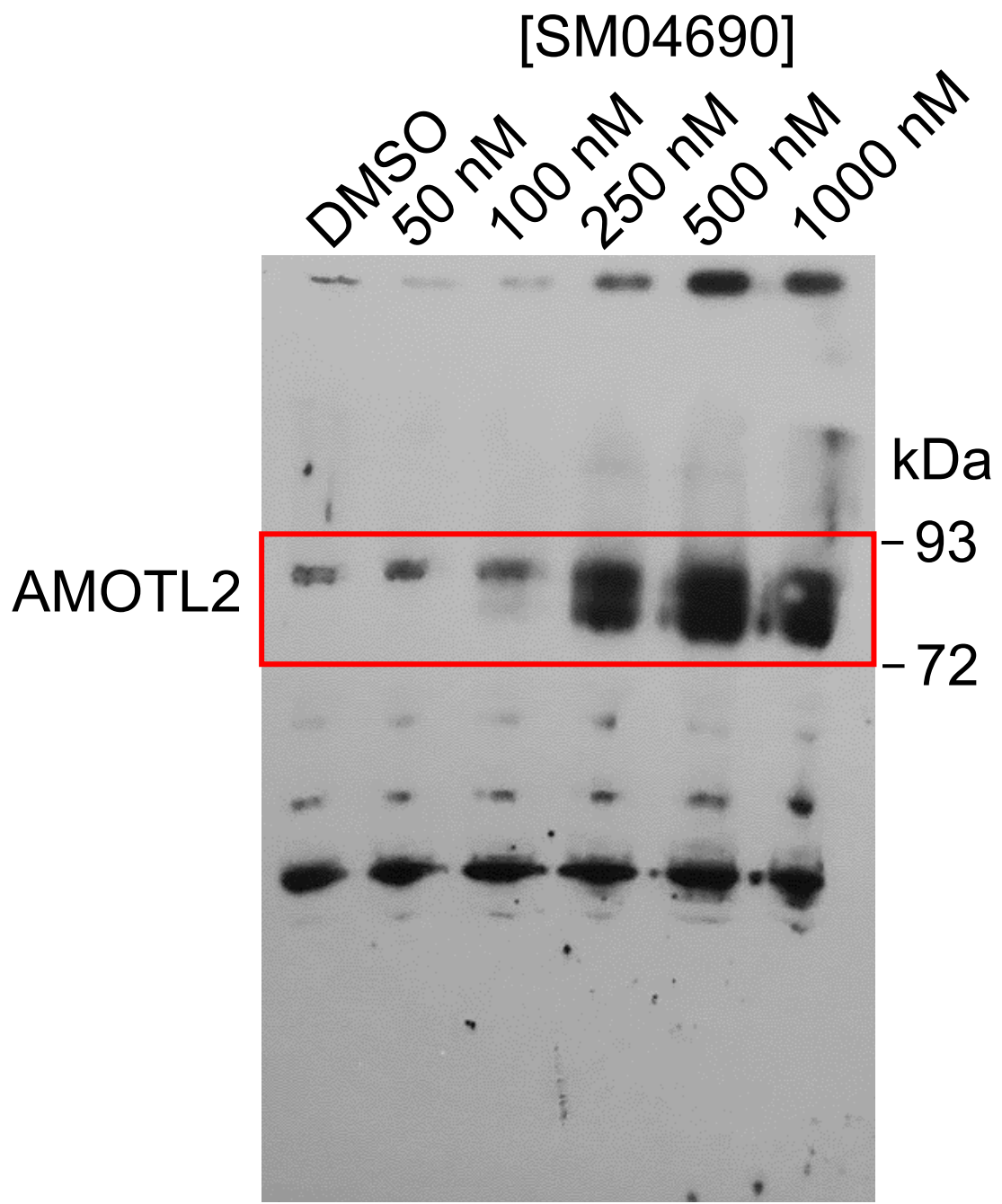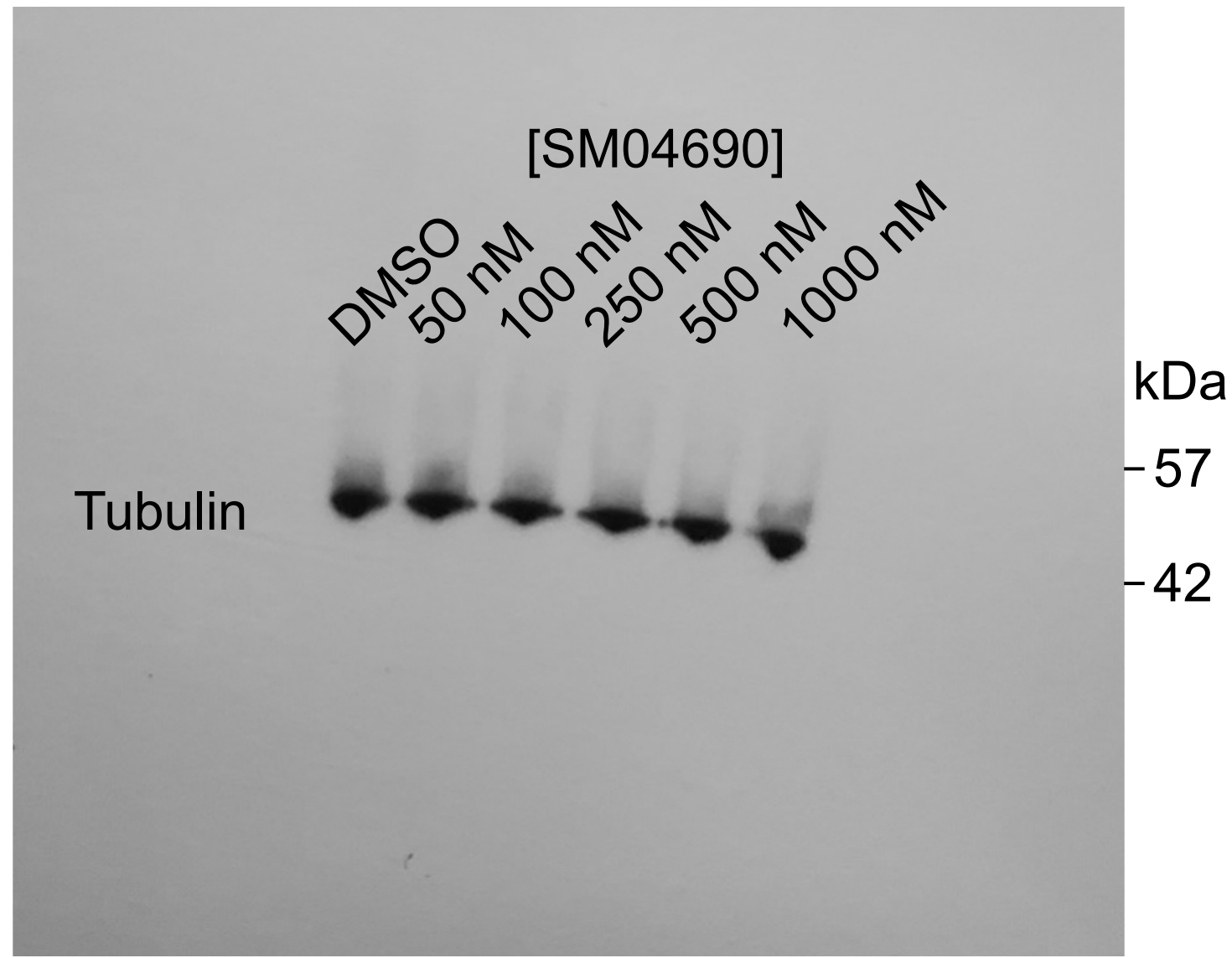

Supplement: Figure 3—source data 2. [file elife-88508-fig3-data2.zip › Figure 3D annotated.pdf]
